# TREATMENT BURDEN AMONG DIVERSE MIDDLE-AGED AND OLDER ADULTS WITH MULTIMORBIDITY

**DOI:** 10.1093/geroni/igae098.3911

**Published:** 2024-12-31

**Authors:** Ana Quiñones, Sheila Markwardt, Anda Botoseneanu, Viet-Thi Tran, Victor Montori

**Affiliations:** Oregon Health & Science University, Portland, Oregon, United States; Oregon Health & Science University, Portland, Oregon, United States; University of Michigan-Dearborn, Dexter, Michigan, United States; Université Paris Cité, Université Paris Cité, Ile-de-France, France; Mayo Clinic, Rochester, Minnesota, United States

## Abstract

The demands associated with ongoing disease management may overwhelm middle aged and older adults living with two or more chronic conditions. Treatment burden refers to both (a) the numerous demands—such as the time and costs of travel to clinical appointment, administrative burden, and coordinating fragmented care—healthcare makes on patients and (b) the negative impact on the patient’s wellbeing. We evaluated data from respondents of the treatment burden experimental module included in the 2022 Health and Retirement Study data collection wave (N=1,888). We analyzed responses to the 15-item Treatment Burden Questionnaire (TBQ; 15 questions scored 0-10; total sum 0-150; >59 considered unsustainable burden). We compared mean total TBQ score by sociodemographic and multimorbidity combination groups using either a t-test (among 2 groups) or ANOVA (among >2 groups). We compared sustainability across groups using a chi-square test. Respondents (mean 69 years; 62% female, 71% living with 2+ chronic conditions) had a mean TBQ score of 20.3/150 (SD 22.7) with 125 (6.6%) reporting unsustainable burden. Administrative and financial tasks and lifestyle changes were the most burdensome activities. Unsustainable burden was significantly more likely among younger (51-64 vs. older), lower educated (< high school vs. ≥high school), and Black and Latino (vs. non-Latino White) respondents, and in those living with 2+ chronic conditions. In this nationally-representative sample of US adults, participants reported less treatment burden than previously reported in international studies. Certain sociodemographic groups reported high and unsustainable burden of treatment, informing future interventions to reduce treatment burden in mid and late life.

